# Postural control during turn on the light task assisted by functional electrical stimulation in post stroke subjects

**DOI:** 10.1038/s41598-022-10893-7

**Published:** 2022-04-29

**Authors:** Andreia S. P. Sousa, Juliana Moreira, Claudia Silva, Inês Mesquita, Augusta Silva, Rui Macedo, Rubim Santos

**Affiliations:** 1 Center for Rehabilitation Research-Human Movement System (Re)Habilitation Area, Department of Physiotherapy, School of Health, Polytechnic of Porto, Rua Dr. António Bernardino de Almeida, 400, 4200-072 Porto, Portugal; 2 Center for Rehabilitation Research-Human Movement System (Re)Habilitation Area, Department of Functional Sciences, School of Health, Polytechnic of Porto, Porto, Portugal; 3 Center for Rehabilitation Research-Human Movement System (Re)Habilitation Area, Department of Physics, School of Health, Polytechnic of Porto, Porto, Portugal

**Keywords:** Neuroscience, Health care

## Abstract

Postural control mechanisms have a determinant role in reaching tasks and are typically impaired in post-stroke patients. Functional electrical stimulation (FES) has been demonstrated to be a promising therapy for improving upper limb (UL) function. However, according to our knowledge, no study has evaluated FES influence on postural control. This study aims to evaluate the influence of FES UL assistance, during turning on the light task, in the related postural control mechanisms. An observational study involving ten post-stroke subjects with UL dysfunction was performed. Early and anticipatory postural adjustments (EPAs and APAs, respectively), the weight shift, the center of pressure and the center of mass (CoM) displacement were analyzed during the turning on the light task with and without the FES assistance. FES parameters were adjusted to improve UL function according to a consensus between physiotherapists’ and patients’ perspectives. The ANOVA repeated measures, Paired sample t and McNemar tests were used to compare postural control between the assisted and non-assisted conditions. When the task was assisted by FES, the number of participants that presented APAs increased (p = 0.031). UL FES assistance during turning on the light task can improve postural control in neurological patients with UL impairments.

## Introduction

Postural control assumes a determinant role in daily life activities, being particularly relevant for those involving large movements of the arm or trunk or when that limb supports the body^1–3^. Being dependent on the continuous afferent information on body position and orientation from visual, vestibular, or somatosensory input and the subsequent motor commands to muscle synergies, postural control is the result of several neural circuits. From these, the supplementary motor area^4,5^, the premotor cortex^6^, and the pontomedullary reticular formation^7–9^ have been demonstrated to have an important role in feedforward mechanisms^10^.

When reaching for an object, feedforward components contribute to motor action optimization [early postural adjustments (EPAs)], posture stabilization [anticipatory postural adjustments (APAs)], weight shift to move the body center of mass (CoM) towards the object, and for the regulation of the CoM position in the base of support [CoM and center of pressure (CoP) relation (CoP-CoM)]^10–15^. In this sense, voluntary movements of a limb are preceded and accompanied by feedforward postural control mechanisms. These prepare the body for the action, and for the expected disturbance of the CoM that will be produced by that movement. Feedforward postural control mechanisms also stabilize the CoM during the execution of the movement itself^10^.

Changes in these postural control mechanisms have been demonstrated in post-stroke subjects. Specifically these changes have been expressed through: (1) decreased APAs in trunk muscles^16,17^; (2) increased trunk compensatory strategies^18^; (3) reduced weight transference for the contralesional limb^19,20^; and (4) deregulation between CoP^21^ and CoM displacement^22,23^. Despite feedforward postural control and voluntary arm movement are thought to be controlled by different pathways, its parallel distribution^7^ has supported the causal relation established between the impairment of feedforward mechanisms and dysfunctional voluntary movement in post-stroke subjects, particularly those that have a lesion in the territory of the middle cerebral artery^24^. In fact, the high percentage of post-stroke patients that present motor control impairments in contralesional limb in reaching tasks can be related to dysfunction of the neural networks that control movement, but also to dysfunction of the neural networks controlling postural control mechanisms^16,17,24,25^.

Previous studies have demonstrated that functional electrical stimulation (FES), applied to the upper limb (UL) muscles, is effective for improving simple single-joint movements, as well as more complex reach-to-grasp movements performed with the contralesional UL in post-stroke patients^26–30^. These findings support the use of FES as a promising therapy in stroke rehabilitation^31^. However, according to our knowledge, no study has assessed the influence of FES assistance in reaching tasks in the related postural control mechanisms. We believe that the cortical reorganization^32,33^ and excitability^34,35^ decurrent from the increased afferent input from muscle spindles and Golgi tendon organs, due to muscle contraction mediated by FES^36^, would contribute to an improvement of postural control. Moreover, based on the evidence that quantitative and clinical measures of postural control improve with task-oriented arm training, without explicit postural control goals, instruction, or feedback^37^, it can be hypothesized that the positive results of UL FES found in post-stroke subjects^31^ could be related to the demonstrated increased movement quality indicators^30,38^, but also to an improvement in postural control mechanisms.

This study aims to evaluate the influence of FES assistance during turning on the light task in the related postural control mechanisms including EPAs and APAs occurrence before the beginning of the task, the weight shift, the regulation of CoM, and CoP forward displacement during reaching. The turn on the light task has been recommended as a real, and daily life purpose task that could be performed by patients with moderate or severe impairment as just involve reaching without grasping^39^.

## Methods

### Subjects

This study is integrated into a more global project with results already published in previous studies^30,38^. A cross-sectional study was performed involving ten subjects (4 females and 6 males), mean age of 53.50 ± 10.97, with a history of a single unilateral stroke (4 ischemic and 6 hemorrhagic), affecting the right (n = 6) and left (n = 4) hemispheres that resulted in a motor control dysfunction of the contralesional UL, with implication in performing turning on the light task, Table 1. Inclusion criteria have already been stated in our previous studies^30,38^ and included: (a) a first unilateral stroke (confirmed by imaging) for at least 6 months; (b) preserved cognitive function, corresponding to a score higher than 23 in the Mini-Mental State Examination (MMSE)^40^ and (c) the ability to perform active movement of the contralesional UL of at least 15° of shoulder flexion and elbow flexion/extension. Exclusion criteria included: (a) hemi-spatial neglect and/or uncorrected visual changes; (b) musculoskeletal or other neurological conditions which might affect ULs and/or trunk function; (c) pain in the ULs; (d) lesion or adverse skin reaction to electrodes gel and/or hypersensitivity to electrical stimulation of the contralesional forearm; (e) epilepsy and frequent convulsions; (f) tumors in the contralesional forearm region submitted to electrical stimulation; (g) clinical signs of increased muscle resistance against passive stretching in the contralesional forearm muscles [confirmed by a score higher than three in the Modified Ashworth Scale (MAS)]; (h) osteosynthesis or metallic implants and/or pacemaker and/or ventriculoperitoneal derivation devices and (i) pregnancy.

**Table 1:** Characterization of post-stroke participants according to sex (M: male, F: female), age (years), weight (kg) and height (m), handedness (R: right, L: left), stroke type (I: ischemic, H: hemorrhagic), sub-type (MCA: middle cerebral artery, ICA: internal carotid artery) and location, injured hemisphere (R; L), time after stroke (months) and the scores of the Mini-Mental State Examination (MMSE), Fugl-Meyer Assessment Scale-Upper Extremity (FMAS-UE), Modified Ashworth Scale (MAS), and Patient Global Impression of Change (PGIC).

ID	Sex	Age (years)	Weight (kg)	Height (m)	Dominance	Type (sub-type)	Location	Injured hemisphere	Time after stroke (months)	MMSE	MAS	FMAS-UE	PGIC
1	M	55	75.0	1.69	L	H (Undetermined)	Thalamo-capsulo-pontine	R	49	28	1 + (2)	47	6
2	M	48	79.0	1.80	R	I (Large artery (MCA))	Fronto-temporo-operculum-insular	L	84	30	1 (1)	42	5
3	M	60	79.0	1.64	R	I (Large artery (Vertebro-basilar))	Lenticulo-capsular	L	56	25	1 (1)	50	3
4	F	42	60.0	1.60	R	H (Undetermined)	Lenticulo-capsular	R	52	30	1	46	3
5	M	40	100.0	1.74	R	H (Undetermined)	Basal ganglia	R	27	27	1 + (2)	28	6
6	M	65	82.0	1.75	R	I (Large artery (MCA))	Fronto-temporo-parietal-insular	R	62	27	2 (3)	39	2
7	M	53	70.0	1.64	R	H (Undetermined)	Lenticulo-capsular	L	63	27	1 (1)	58	3
8	F	64	60.0	1.59	R	H (Undetermined)	Thalamo-capsular	L	37	23	1 + (2)	40	3
9	F	39	80.0	1.67	R	H (Undetermined)	Striato-capsular	R	141	29	1 + (2)	33	3
10	F	69	66.0	1.46	R	I (Lacunar)	Lenticulo-capsular	R	12	27	0 (0)	41	6
M ± SD	4F;6 M	53.50 ± 10.97	75.10 ± 11.96	1.66 ± 0.10	9R;1L	4I;6H	_	6R;4L	58.30 ± 35.35	27.30 ± 2.16	–	42.40 ± 8.50	4.10 ± 1.45

### Ethical considerations

Ethical approval was obtained by the local and the national Ethics Committee for Clinical Research (CEIC). The study was also approved by INFARMED I. P., and was registered at ClinicalTrials.gov with identifier: NCT03967613. All participants gave written informed consent before the data collection began as per the Declaration of Helsinki.

### Instruments

The Portuguese version of Mini-Mental State Examination (MMSE)^40,41^, Fugl-Meyer Assessment Scale-Upper Extremity (FMAS-UE)^42^, and Modified Ashworth Scale (MAS)^43^ were applied to assess basic cognitive functions^40^, UL sensorimotor impairment severity^44^, and the muscle resistance against passive stretching in the contralesional forearm muscles^43^, respectively. The Portuguese version of the Patient Global Impression of Change (PGIC) scale^45^ was used to assess the participants’ perception of change concerning the movement of the contralesional UL assisted by FES against without stimulation. Height and weight were obtained using a stadiometer (seca^®^ 222, Seca GmbH & Co. KG, Hamburg, Germany) and a balance scale (seca^®^ 760).

**Figure 1:**
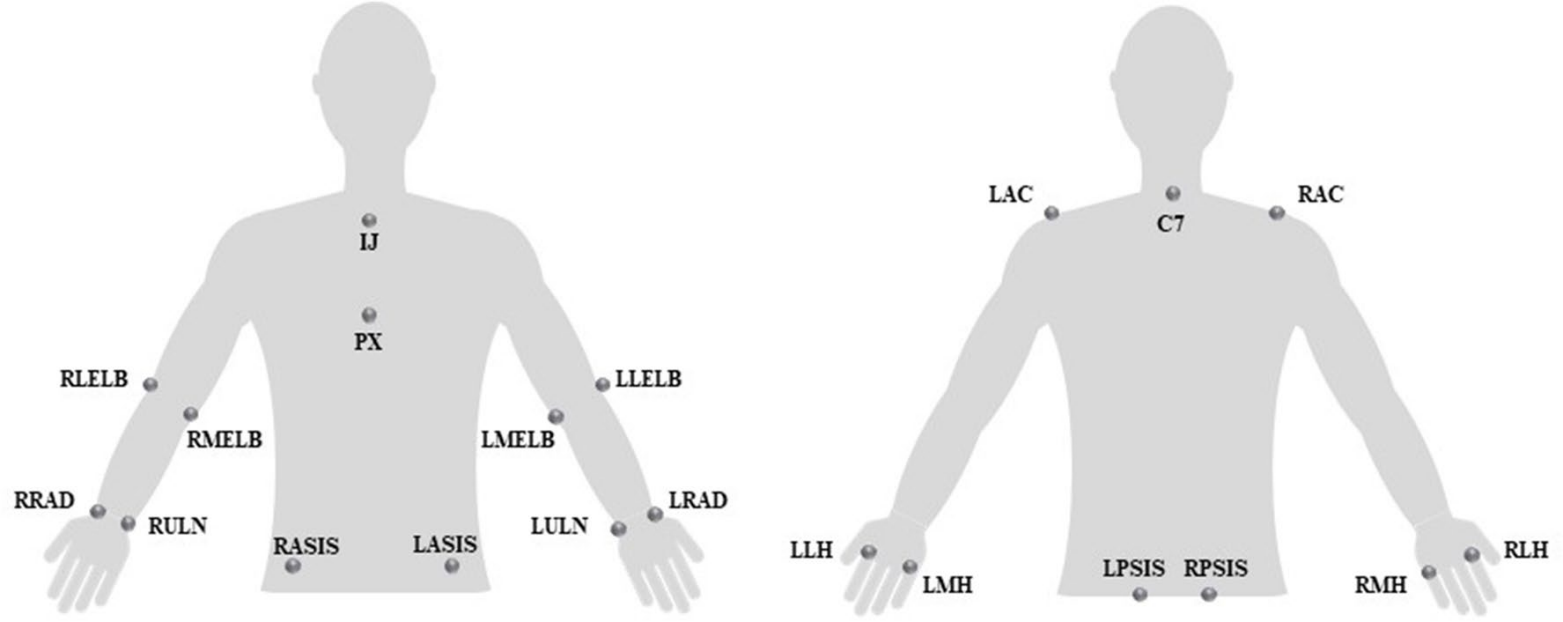
Anatomical references for reflective markers placement. *C7*spinous process of the 7th cervical vertebra; *IJ* incisura jugularis, *LAC* middle part of left acromion, *LASIS* left anterior superior iliac spine, *LLELB* lateral epicondyle of left humerus, *LLH* lateral side of the head of the second left metacarpal, *LMELB* medial epicondyle of left humerus, *LMH* medial side of the head of the fifth left metacarpal, *LPSIS* left posterior superior iliac spine, *LRAD* styloid process of left radius, *LULN* styloid process of left ulna, *PX* processus xiphoideus, *RAC* middle part of right acromion, *RASIS* right anterior superior iliac spine, *RLELB* lateral epicondyle of right humerus, *RLH* lateral side of the head of the second right metacarpal, *RMELB* medial epicondyle of right humerus, *RMH* medial side of the head of the fifth right metacarpal, *RPSIS* right posterior superior iliac spine, *RRAD* styloid process of right radius, *RULN* styloid process of right ulna.

The kinematic data of the trunk and contralesional UL and ground reaction forces were acquired during the performance of the functional task through Qualisys System (Qualisys AB^®^, Gothenburg, Sweden), with eight Oqus cameras (operating at 200 Hz) and a set of 15 reflective markers (Fig. 1). The values of the vertical (Fz) component of ground reaction forces (GRF), as well as the values of center of pressure in anteroposterior (CoP_AP_) and mediolateral (CoP_ML_) directions, were acquired using two force plates at a sampling rate of 100 Hz (FP4060-08 and FP4060-10 models from Bertec Corporation (USA), connected to a Bertec AM 6300 amplifier and to an analog board, from Qualysis, Inc. (Sweden)). The Visual 3D Professional software, version 6 was used to perform all the events detections and metric calculations.

**Figure 2:**
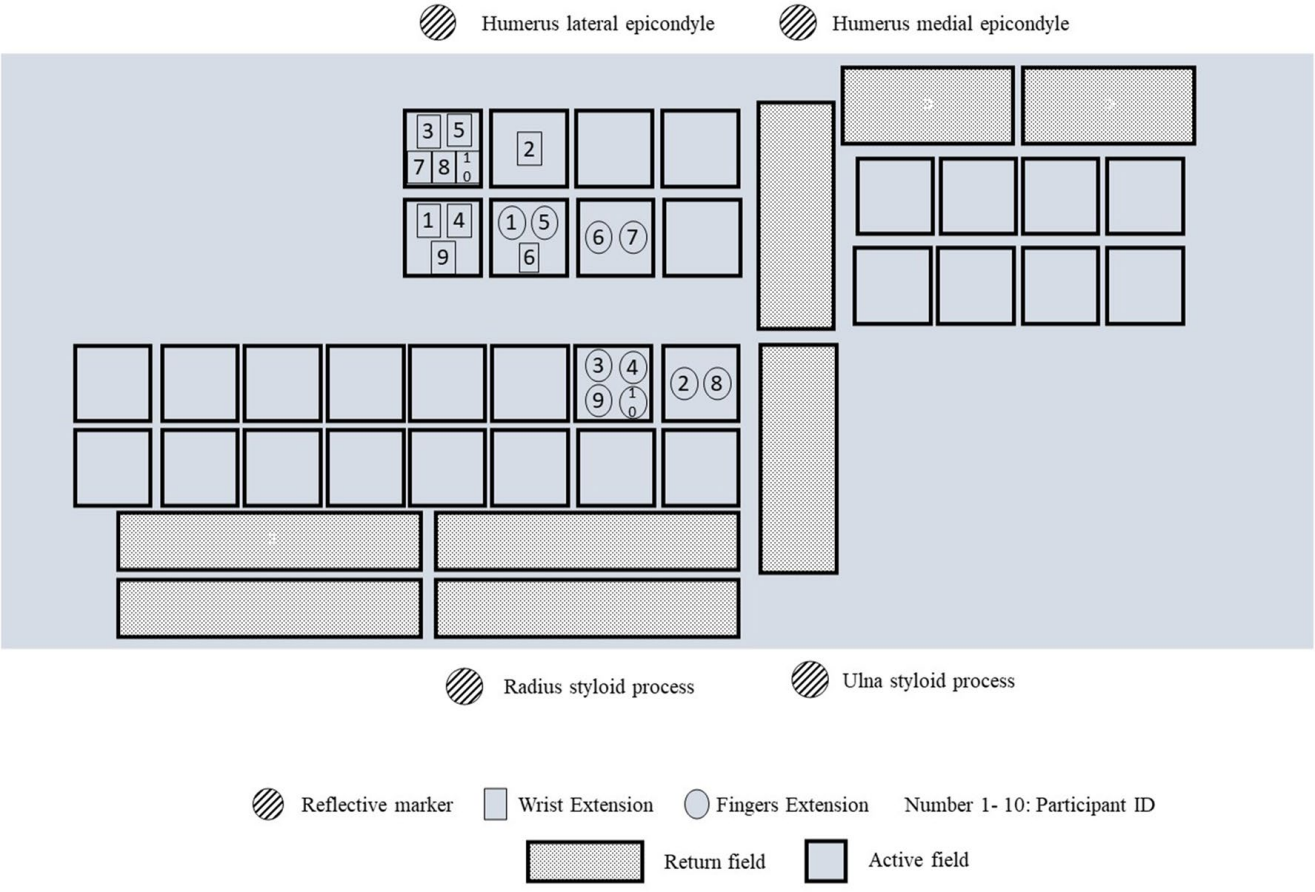
Representation of the active fields selected for each patient.

A superficial multi-field electrode FES system (FES-HAND, Tecnalia Research & Innovation-Health Division, San Sebastián, Guipúzcoa, Spain) was used. The FES-HAND system includes a 40 channels’ stimulator device, with 32 cathodes or active fields and 8 anodes or return fields, and a multi-field electrode supported by a textile garment covering the forearm. The stimulator had a predefined frequency of 35 Hz, a pulse width of 300 µs (biphasic symmetrical), and a begin/end ramp time of 1 s. The stimulation fields, the intensity, and the stimulation time were configured through a dedicated software application developed for this purpose (NeuroClinic FES v1.9.24). The number active fields, the intensity of each active field and the time of the active fields were adjusted for each patient to obtain a selective motor response regarding wrist and fingers extension movements. A representation of the active fields selected for each patient is presented in Fig. 2.

All subjects used standard tennis footwear (1.5 cm heel), in their adequate size, as different footwear leads to divergent levels of postural stability, reflected in CoP displacement^46^.

### Procedures

Before the evaluation, each participant took part in five adaptation sessions to the electrical stimulation in the contralesional limb. These sessions involved a randomized stimulation for ten minutes to familiarize the patient with the sensation produced by the application of FES and to increase motor nerve excitability. In the first adaptation session, the stimulation intensity was increased from 5 mA to a comfortable motor threshold, maintained for 1–2 min, with further increases of 2 mA according to the patient’s tolerance. In the subsequent sessions, the protocol was repeated, but the stimulation intensity started with a value of 5 mA lower than the maximum amplitude reached in the previous session. After this period, an expert physiotherapist manually tested and defined the active electrode fields, intensities, and stimulation time leading to a selective motor response regarding wrist and fingers extension movements^47–49^ and improving UL function during the tuning on the light for each patient (Fig. 2). It was considered that the function was improved when a score higher than 3 was obtained on the PGIC scale. The stimulation parameters saved in the last randomized stimulation session were always tested and manually adjusted by the physiotherapist before the kinematic evaluation of the task. No adverse effect occurred, nor any subject complaint with the stimuli.

The participants were requested to turn on the light by pressing a switch with the contralesional limb from a sitting position and at a comfortable self-selected speed. The seat height was adjusted to 100% of the leg length (measured from the lateral epicondyle of the femur to the ground). The task was performed without trunk support or restraint, with three-fourths of the femur length supported, and each foot positioned in each force plate^50^. The switch (42.25 cm^2^ of area) was attached to a wooden board containing a lamp and an electrical circuit allowing it to light up once the switch was pushed. All the system with the lamp was placed on a table located in front of each participant. The height of the table was adjusted to the olecranon’s height and the switch was placed in front of the ipsilateral hip (sagittal plane) at a distance of this joint equal to the length between the acromion and the trapezium-metacarpal joint of the ipsilateral UL^51^. The participants performed six trials of the task, three with and three without the assistance of FES (FES-HAND). When the task was assisted by FES-HAND in the contralesional limb, the patients were instructed to actively participate in the movement after feeling the electrode stimulus. When the task was performed without FES-HAND assistance, the participants were verbally informed when to start the task.

### Data processing

#### Event definition of task phases detection

The kinematic data was processed through Qualisys Track Manager (Qualisys AB, Gothenburg, Sweden) and Visual3D (C-Motion, Inc., Germantown, USA) software following the International Society of Biomechanics recommendations and the methods of previous studies^52^. The movement trajectory and force plate data were low-pass Butterworth filtered with a cut of frequency of 6 Hz and 20 Hz, respectively.

The “onset” of the task, designated by T0, was defined as the time when the tangential velocity of the hand exceed 2% of the maximum velocity in the reaching phase^53^. The “reaching” phase end (beginning of the return to start position) was defined as the instant when the linear velocity of the hand crossed the zero value downwards in the sagittal plane.

#### Postural control metrics

**Figure 3:**
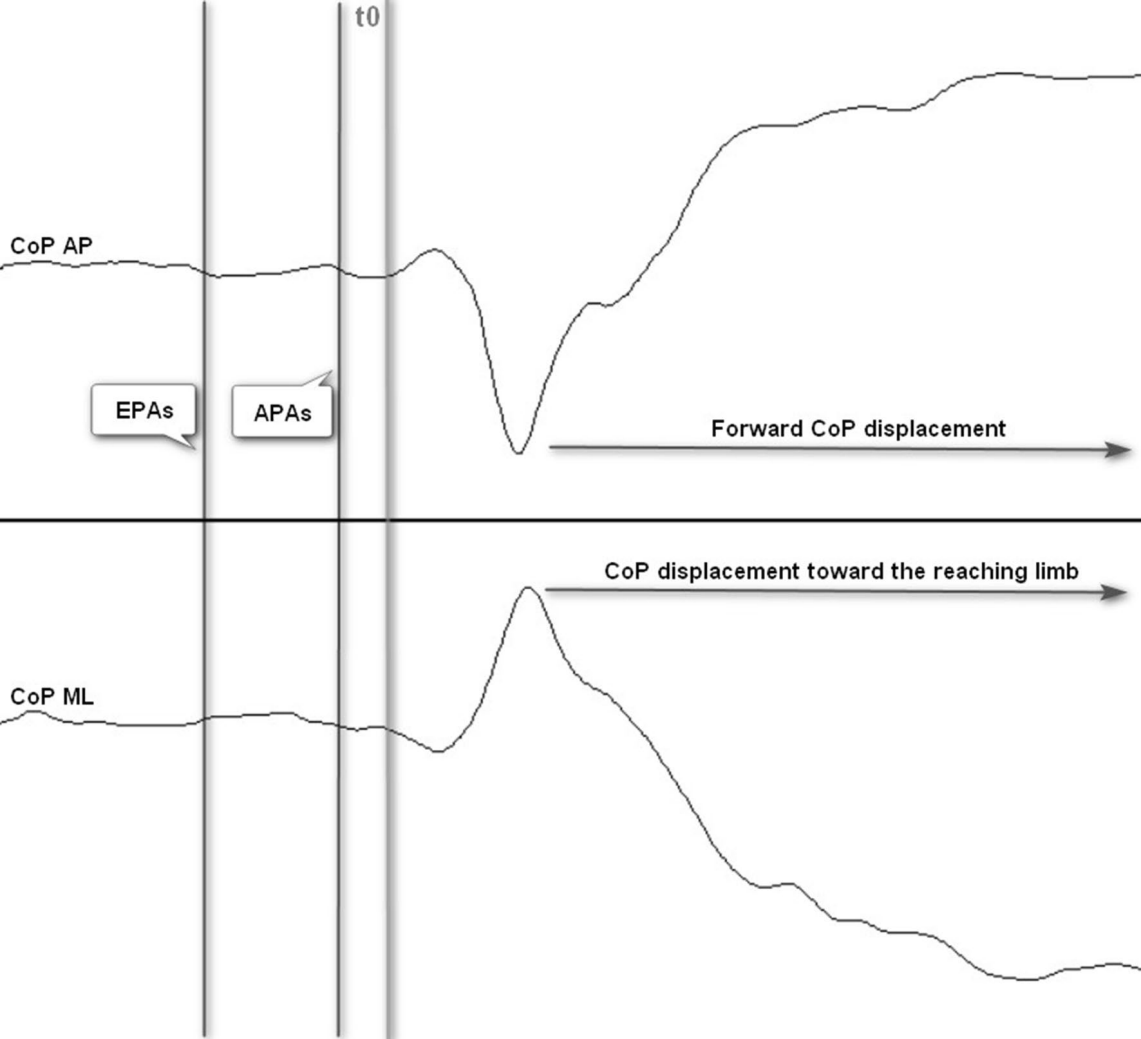
Representation of EPAs and APAs timing and CoP displacement during reaching when the task was assisted by FES-HAND.

The CoP_AP_ backward displacement in the time window of early postural adjustments (EPAs) (from 600 to 250 ms before T0^15^) and anticipatory postural adjustments (APAs) (from 200 ms before to 50 ms after T0^54^) was defined as an interval lasting for, at least, 50 ms when its value was lower than the mean minus 3 standard deviations (SD) of the baseline of the center of pressure displacement in the anteroposterior direction (CoP_AP_). The baseline of the center of pressure displacement in the direction of the contralesional limb (CoP_ML_) was defined as an interval lasting for, at least, 50 ms when its value was lower or higher than the mean minus or plus 3 SD of its baseline, respectively, depending on if the contralesional limb was the left or right limb. The baseline interval for EPAs was considered from 650 to 600 ms before T0^15^, and for anticipatory postural adjustments was considered from 250 to 200 ms before T0^54^. If the participant presented a backward displacement or a displacement in the direction of the contralesional limb in the time window of EPAs or APAs, it was considered the participant presented EPAs or APAs, respectively (Fig. 3). It was considered for analysis the number of participants that presented EPAs or APAs, and EPAs and APAs.

The CoM and CoP displacement, during the reaching phase, was calculated as the difference between the end and the beginning of the reaching phase in the anteroposterior and mediolateral directions (Fig. 2). The difference between CoM and CoP (CoP-CoM) was calculated to assess postural stability during the reaching phase^22^. The Asymmetry Index was calculated^55^ to provide a measure of the amount of weight-bearing variation on each limb during reaching:

$$Asymmetry\ Index=\frac{({\Delta Fy}_{CONTRA}-\Delta {Fy}_{IPSI})}{(0.5({\Delta Fy}_{CONTRA}+{\Delta Fy}_{IPSI}))}\times 100,$$

where $\Delta Fy$ refers to the vertical ground reaction force variation during reaching. A higher Asymmetry Index score represents greater weight-bearing on the contralesional limb while ‘0’ represents perfect symmetry (50% weight-bearing on each limb), during reaching phase of turning on the light. The arithmetic mean of three valid trials was used for the analysis.

### Statistics

Version 25.0 of the Statistical Package for the Social Science (SPSS^®^) software was used for descriptive and inferential statistical analysis, with a level of significance of 0.05.

The ANOVA repeated measures was used to compare the CoM and CoP displacement variables and the CoP-CoM differences, considering the AP and ML directions as well the composed value during the reaching phase of the turning on the light task, between the assisted and non-assisted conditions. The paired sample t test was used to compare the Asymmetry Index during the same phase between conditions. The McNemar test was used to compare the proportion of participants that presented early postural adjustments and/or anticipatory postural adjustments between the task performed with and without FES-HAND assistance.

## Results

**Figure 4:**
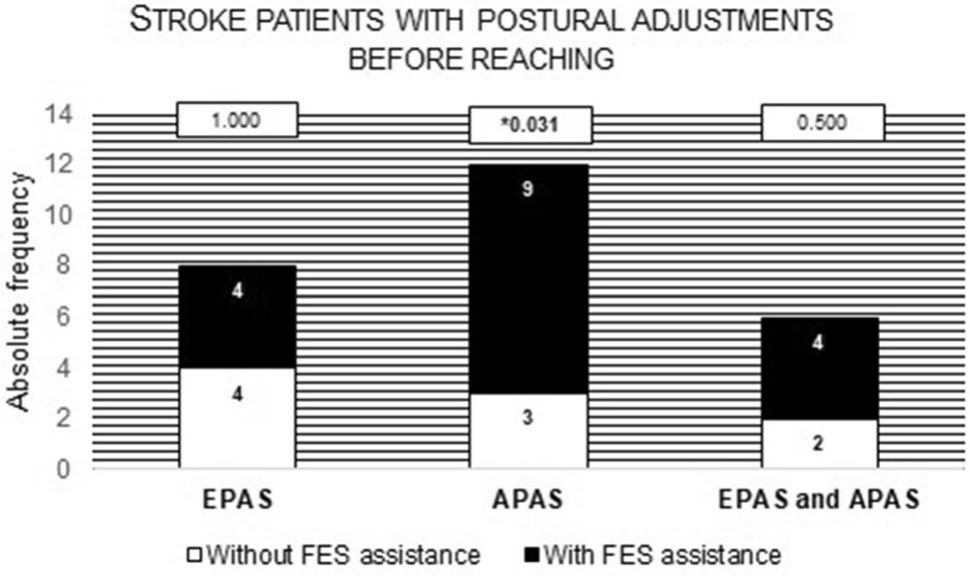
Description of the number of participants that presented EPAs or APAs and EPAs and APAs before the beginning of the turning on the light with and without FES-HAND assistance. Proof values (p values) from between conditions comparisons are presented. Statistically significant values were identified in bold and with *.

As observed in Fig. 4, statistically significant differences were noted in the number of participants that presented APAs between conditions (p = 0.031, (1 − β) = 0.75). Specifically, when the task was performed without assistance, APAs were only observed in three participants. When the task was performed with FES-HAND assistance, APAs were observed in the same three participants but also in more 6 participants, performing a total of 9 participants. Also, the number of participants that presented both EPAs and APAs duplicated when the task was assisted by FES-HAND. However, the differences were not statistically significant (p = 0.500, (1 − β) = 0.63) (Fig. 4).

**Figure 5:**
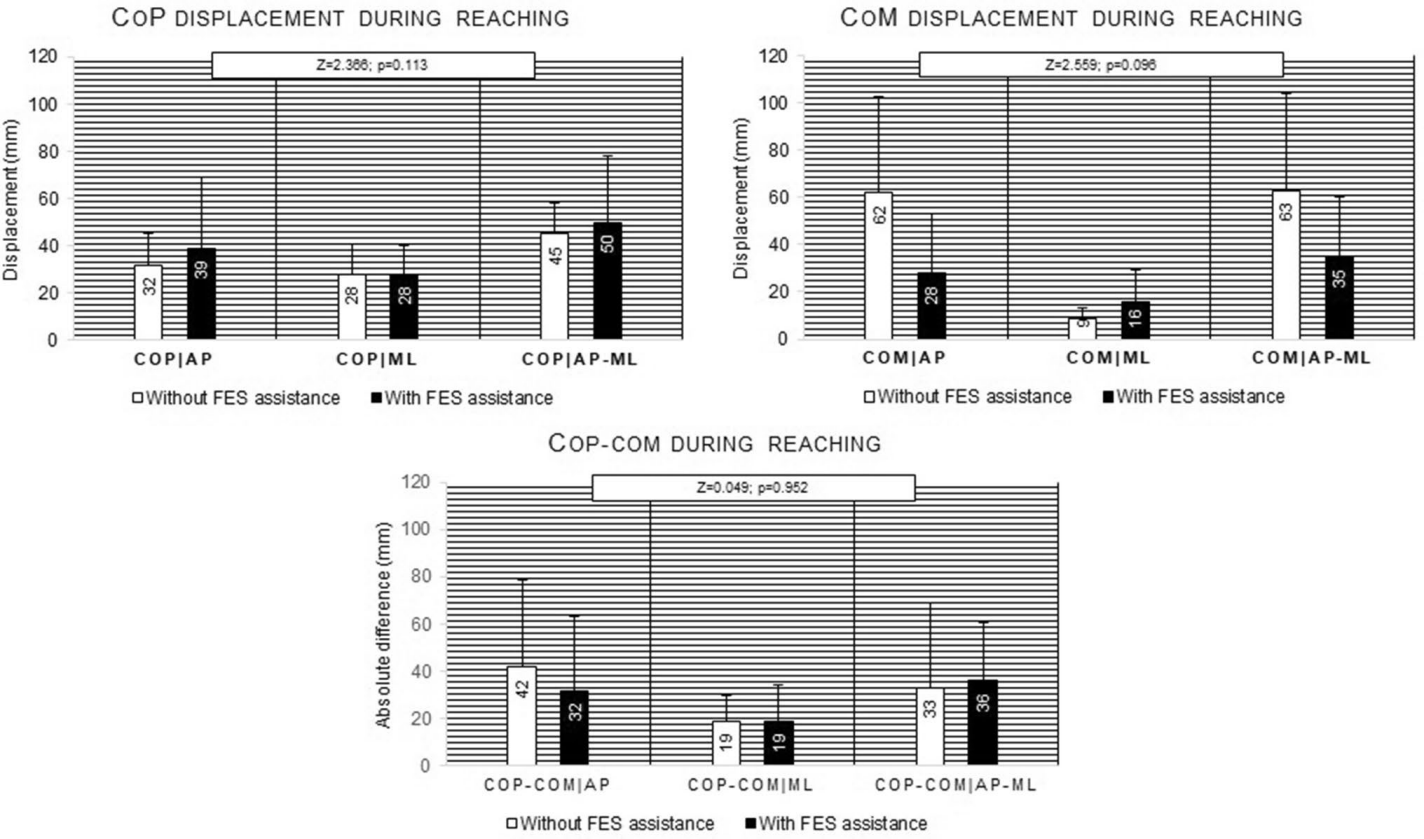
Mean and standard deviation values of displacement of CoP, CoM, and CoP-CoM for AP, ML, and AP-ML directions during the reaching phase of turning on the light with and without FES-HAND assistance. Between conditions comparisons p values are presented. Statistically significant values were identified in bold and with *.

Figure 5 present the values of CoP and CoM displacement, during the reaching phase of the turning on the light performed, with and without FES-HAND assistance. While a tendency to decreased CoM_AP,_ combined with an increase of CoP_AP_ and decrease of the related difference was observed when the task was assisted by FES-HAND (Fig. 5), no statistically significant differences were observed in CoP (Z = 2.366, p = 0.113, (1 − β) = 0.437) and CoM displacement (Z = 2.559, p = 0.096, (1 − β = 0.467), as well in CoP-CoM difference (Z = 0.049, p = 0.952, (1 − β) = 0.057).

**Figure 6:**
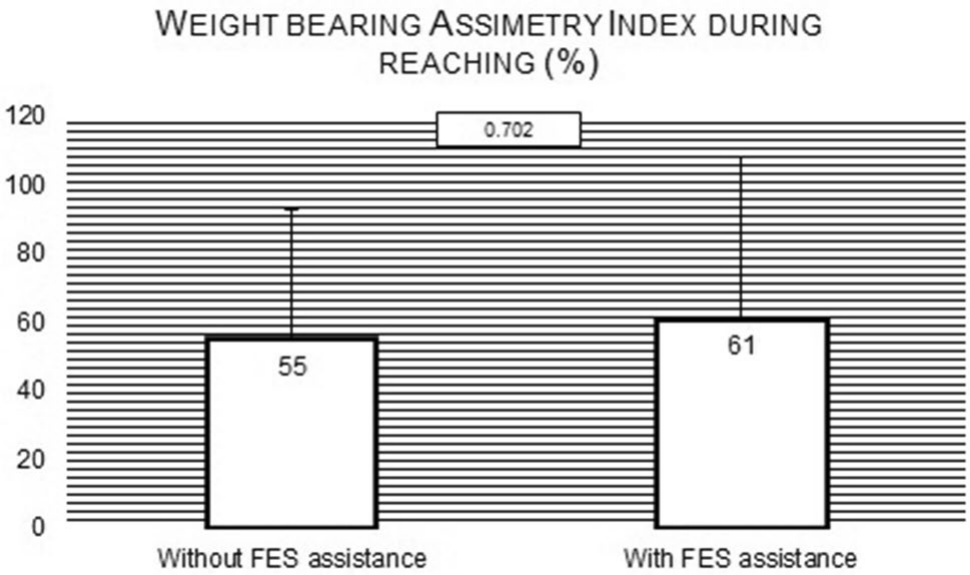
Mean and standard deviation values of weight-bearing Asymmetry Index during the reaching phase of turning on the light with and without FES-HAND assistance. Between conditions comparisons p values are presented.

Despite a tendency to an increase of weight-bearing Asymmetry Index toward the contralesional limb when the task was assisted by FES-HAND (Fig. 6), the differences were not statistically significant (t = − 0.395, p = 0.702, (1 − β) = 0.138).

## Discussion

The results obtained in the present study demonstrate that when turning on the light was assisted by FES-HAND, the number of participants that presented APAs increased from 3 to 9. The APAs were associated with a displacement of CoP backward or toward the contralesional limb which is probably resultant from increased APAs in trunk muscles^56–59^ and less likely to result from changes in APAs in leg muscles. In fact, since the task was performed from a sitting position, the APAs in leg muscles are attenuated as a consequence of: (1) a large base of support turning the task of maintaining the CoM projection within boundaries of the base of support less challenging; (2) a closer position of the CoM to the base of support; (3) different inertia values because the lower part of the body is supported when sitting. The increased number of participants that presented APAs seems to be a positive results as decreased APAs in trunk muscles have been described in stroke patients^16,17^ and increased use of the trunk during reaching^60–62^ may limit recovery of independent movements of the affected arm of stroke patients^18^.

The positive findings over global postural control parameters, decurrent from a contralesional focal movement assistance with FES-HAND, can be explained by the activation of a proprioceptive map in the contralesional side. In fact, FES leads to the recruitment of afferent receptors that modulate spinal and cortical circuits^63,64^. The FES training by increasing afferent input from muscle spindles and Golgi tendon organs, due to muscle contraction^36^, causes cortical reorganization^32,33^ and somatosensory inputs leading to changes in the cortical excitability^34,35^. Specifically, it has been demonstrated that FES increases the excitability of areas closely related to postural control mechanics during the preparation and initiation of movement and its correction^65^, as supplementary motor areas^66^ and cerebellum^67^.

The improvement of postural control provided by FES-HAND assistance observed in the present study could be associated with our previous findings demonstrating increased UL movement quality when reaching tasks were assisted by FES-HAND^29,30^. In fact, feedforward postural control and voluntary arm movement descending pathways^7^ needed to be integrated for effective activity completion^68^ without loss of postural control. In this perspective, our results seem also to suggest that the increased mechanical, sensory-motor information provided by FES-HAND facilitates an implicit learning process for the postural control (see Pohl et al.^69^ for details on implicit learning after stroke). It is important to highlight that the FES parameters were adjusted up to the patient participants attribute a score higher than 3 on the PGIC scale. The results obtained demonstrated that the score attributed ranged from 3 (*a little better, but no noticeable change)* to 6 (*better, and a definitive improvement that as made a real and worthwhile*), indicating the FES parameters should be adapted to each patient needs.

Despite FES improved APAs in 60% of the participants, it should be considered that it seemed not to influence some participants, as well other postural control variables as EPAs and those related to the reaching phase as CoP and CoM displacement, and weight shift. During turning on the light task, a weight shift is needed to move the CoM toward the target^11,12^, while CoP displacement is needed as counterbalancing procedure in response to the reaching arm^70,71^. Previous studies have demonstrated that post-stroke subjects have difficulty transferring weight for the contralesional limb, particularly when the task is performed with that limb^19,20^. Additionally, post-stroke subjects present a larger CoP displacement in the lateral direction when reaching straight forwards^21^, exceeding the CoM displacement^22,23^. Some factors probably contributed to the lack of influence of the FES-HAND assistance over these variables. First, in the present study, only the immediate effect of FES-HAND system was assessed. We believe that, with FES-HAND assisting training sessions, more participants would develop APAs. Second, it should be considered the setup used to perform the task, as well the force plates location. In fact, in seated postural control, the trunk mass has to stay within the base of support defined by the buttocks and feet. However, the EPAs and APAs, CoP displacement, and weight shift were searched from the signals of two force plates located only below the feet. It can be argued that a force plate below the buttocks would probably be more sensible for detecting the CoP displacement and weight shift changes when the task was assisted by FES-HAND. In this perspective, future studies analyzing the impact of FES-HAND over postural control mechanisms detected with a force plate located below the buttocks are required to confirm the inexistence of influence of FES-HAND over the mentioned postural control variables.

The results of the present study demonstrated a positive immediate effect of UL FES assistance in postural control variables. We believe that this effect would be more pronounced after FES-HAND assisting training sessions, however future studies assessing the effect of FES-based therapy over postural control parameters and its relation with clinical scores are needed to confirm this idea.

## Conclusion

The findings obtained in the present study demonstrate that FES assistance improves APAs, as the number of participants that presented this postural adjustment increased.
